# Zu hoch geschätzte Prävalenz von lebensbedrohlichen und lebensverkürzenden Erkrankungen bei Kindern und Jugendlichen in Deutschland – Kommentar zum Artikel von Burgio und Jennessen (Ausgabe 7/2023)

**DOI:** 10.1007/s00103-023-03789-y

**Published:** 2023-11-16

**Authors:** Holger Hauch, Maria Janisch, Boris Zernikow, Claudia Bausewein

**Affiliations:** 1 Kinderpalliativzentrum an der Vestischen Kinder- und Jugendklinik, Universität Witten/Herdecke, Datteln, Deutschland; 2 Lehrstuhl für Kinderschmerztherapie und Pädiatrische Palliativmedizin, Fakultät für Gesundheit, Department für Humanmedizin, Universität Witten/Herdecke, Witten, Deutschland; 3 AG Kinder und Jugendliche der Deutschen Gesellschaft für Palliativmedizin, Deutsche Gesellschaft für Palliativmedizin e. V., Aachener Str. 5, 10713 Berlin, Deutschland; 4 Sächsisches Kinderpalliativzentrum, Universitätsklinikum Dresden, Carl Gustav Carus Universität, Dresden, Deutschland; 5 Klinik und Poliklinik für Palliativmedizin, LMU Klinikum, LMU München, München, Deutschland

## Allgemeines

Wir möchten zunächst einen großen Dank an die Verfasser:innen der PraeKids-Studie (Erhebung der Prävalenz von Kindern und Jugendlichen mit lebenslimitierenden und lebensbedrohlichen Erkrankungen in Deutschland) aussprechen. Es ist sehr zu begrüßen, dass erste Daten zur Berechnung von Häufigkeiten lebensbedrohlicher oder lebensverkürzender Erkrankungen auch in Deutschland vorgelegt werden.

Die genaue Anzahl von Kindern und Jugendlichen mit einer lebensbedrohlichen oder lebensverkürzenden Erkrankung ist in Deutschland nicht bekannt. Bislang wurde aufgrund von Ableitungen der Studie von Fraser et al. [1] für die Jahre 2009/2010 in England geschätzt, dass von etwa 50.000 Betroffenen in Deutschland auszugehen sei. Aktuelle Untersuchungen von Fraser et al. zeigen aber eine deutliche Zunahme der Prävalenz von 238 pro 100.000 in den Jahren 2001/2002: (95 % Konfidenzintervall [KI]: 236–241) auf 664 pro 100.000 in den Jahren 2017/2018 (95 % KI: 660–668). Unter Berücksichtigung dieser aktuellen Daten müsste man von ca. 100.000 Kindern und Jugendlichen in Deutschland ausgehen [2].

Die Ergebnisse der PraeKids-Studie gehen für den Untersuchungszeitraum zwischen 2014 und 2019 von einer 3‑ bis 11-mal höheren Prävalenz von Kindern und Jugendlichen mit lebensbedrohlichen und/oder lebenslimitierenden Erkrankungen in Deutschland aus. Wir halten es für sehr wichtig, auf die aus unserer Sicht viel zu hoch geschätzte Prävalenz hinzuweisen, weil damit Bedarfe für diese vulnerable Gruppe unserer Gesellschaft formuliert werden könnten, die in dieser Form nicht bestehen. Nachfolgend legen wir dar, welche Faktoren diese sehr unterschiedlichen Ergebnisse erklären könnten.

Die PraeKids-Studie verwendet Abrechnungsdaten von gesetzlichen Krankenkassen aus den Jahren 2014 bis 2019, die vom Spitzenverband der gesetzlichen Krankenkassen (GKV-SV) und dem Institut für angewandte Gesundheitsforschung (InGef) zur Verfügung gestellt wurden. Um die Prävalenz lebensbedrohlicher und/oder lebensverkürzender Erkrankungen zu ermitteln, wurden anhand von Stichproben Häufigkeiten von zuvor festgelegten ICD-10-Kodierungen berechnet.

Je nach verwendeter Datengrundlage und Art der ICD-10-Filterung ergaben die Berechnungen von Burgio und Jennessen, dass im untersuchten Zeitraum 319.948 bis 402.058 Kinder und Jugendliche an lebensbedrohlichen und/oder lebensverkürzenden Erkrankungen litten. Dies entspräche einer bundesdeutschen Prävalenz zwischen 2.539/100.000 (2014; GKV) und 2.622/100.000 (2019; GKV). Im Vergleich dazu zeigten die bereits genannten Ergebnisse von Fraser et al. Prävalenzen von zuletzt 664/100.000 (für die Jahre 2017/2018). Ähnliche Untersuchungen in Schottland kamen auf eine Prävalenz der stationären Fälle von 414/100.000 für die Jahre 2013/2014 [3]. Es ist anzunehmen, dass der Unterschied in den Prävalenzen noch größer wäre, wenn Burgio und Jennessen nicht nur Kinder und Jugendliche im Alter zwischen 0–19 Jahren eingeschlossen hätte, sondern wie Fraser et al. bei der schottischen Studie [3] Betroffene im Alter von 0–25 Jahren.

Aus unserer Sicht sind folgende Punkte zu diskutieren, von denen wir uns gewünscht hätten, dass diese auch in der publizierten Originalarbeit bereits erwähnt worden wären.

## Grundlegende Unterschiede in der Methodik

Burgio und Jennessen verwendeten als Grundlage für die ICD-10-Filterungen eine Stichprobe der Abrechnungsdaten von (im Jahr 2019) 109 gesetzlichen Krankenversicherungen. In der größten Gruppe der GKV-Patient:innen wurden ausschließlich ambulante Abrechnungsdaten ausgewertet.

Fraser et al. griffen auf Daten des staatlichen und steuerfinanzierten National Health Service (NHS) zurück und analysierten ausschließlich stationäre Behandlungen, u. a. weil die Datenqualität aus den ambulanten Fällen als nicht ausreichend erachtet wurde. Außerdem ist hypothetisch davon auszugehen, dass stationär behandelte Kinder und Jugendliche schwerer erkrankt sein dürften als rein ambulant Behandelte. Dazu schreiben Burgio und Jennessen: „Hinsichtlich der Falldefinition wurden Patient:innen mit jeweils mindestens einer stationären Haupt- oder Nebendiagnose *und/oder *2 gesicherten ambulanten Diagnosen in verschiedenen Quartalen des jeweiligen Analysejahres (M2Q-Kriterium) berücksichtigt.“ Das bedeutet, dass es auch bei InGef ausschließlich ambulante Fälle geben müsste. Auch Burgio und Jennessen untersuchten – basierend auf den InGef-Daten – auch stationäre Falldaten, ohne dass diese gesondert der Originalarbeit oder dem ausführlichen Studienbericht zu entnehmen gewesen wären.

Ferner wählten Burgio und Jennessen im Gegensatz zur Totalerhebung des NHS die Vorgehensweise, eine Stichprobe basierend auf wechselnden, insgesamt aber lediglich 7 Geburtstagen/Jahr zu verwenden. Es sei 1 Geburtstag/Abrechnungsjahr verändert worden. Wir halten das Vorgehen von Burgio und Jennessen für problematisch, weil es zu Verzerrungen kommen könnte, die die Repräsentativität der Stichprobe infrage stellen.

## Repräsentativität der Stichprobe

Die Geburtenrate Deutschlands weist erhebliche Schwankungen auf (im Zeitraum der PraeKids-Studie zwischen 53.344 und 73.589 Geburten/Monat [4]), die sich verzerrend auf die Prävalenzberechnungen auswirken könnten. Gemäß den Beschreibungen der Methodik der Stichprobenziehung bei Burgio und Jennessen wäre der Stichprobenumfang wie folgt zu berechnen:

$$\text{Zahl der Versicherten }< /=19\text{ Jahre }(=12.391.184);$$

$$12.391.184\times 7/365{,}25(\text{Faktor})=237.476.$$

(Anmerkung: Der Faktor ergibt sich aus 7 Geburtstagen/Analysejahr (inkl. Berücksichtigung eines Schaltjahres im Untersuchungszeitraum).)

Diese Zahl ist ein Schätzwert, da nicht sicher davon ausgegangen werden kann, dass geburtsstarke und geburtsschwache Tage der Stichprobe sich automatisch ausgleichen. Hinzu kommt, dass die Stichprobengröße von 7/365,25 sehr seltene Erkrankungen ggf. nicht detektieren könnte. In der vorliegenden Publikation wird beschrieben, dass der jeweils Erste eines Monats als Geburtstag aus der GKV-Stichprobe nicht aufgenommen werden konnte. Dies könnte gerade bei geflüchteten Kindern – u. a. wegen differenter Kalenderformate (z. B. gregorianischer Kalender: 01.01.2014 = 28 Safar 1435 im islamischen Kalender) zu weiteren Verzerrungen in der Studienpopulation geführt haben, da bei geflüchteten Kindern der 01.01. des Geburtsjahres sehr häufig als Geburtstag angegeben wird. Aus der Drucksache 19/29924 des Deutschen Bundestages ist zu entnehmen, dass beispielsweise in Thüringen im Jahr 2016 21,0 % der Asylsuchenden den 01.01. als Geburtstag angegeben haben [5].

In der Betrachtung der onkologischen Diagnosen verdichten sich die Hinweise, dass die verwendete Stichprobe in der PraeKids-Studie nicht repräsentativ sein dürfte. Die Daten von Burgio und Jennessen stehen im Widerspruch zu den Daten des Deutschen Kinderkrebsregisters, in dem seit mehr als 40 Jahren Daten zu onkologischen Diagnosen zusammengeführt werden. So erkranken laut Kinderkrebsregister in allen Altersjahrgängen mehr Jungen als Mädchen (z. B. 2018: Gesamtzahl an Neuerkrankungen = 2255, davon 1215 Jungen [6]). Auch international wurde eine eindeutige Knabenwendigkeit (männlich/weiblich 1,3/1) bei kindlichen Krebserkrankungen mehrfach beschrieben [7]. Hingegen zeigen die Daten der PraeKids-Studie bei onkologischen Erkrankungen ein ausgeglichenes Geschlechterverhältnis (Abb. 4 im online verfügbaren Studienbericht [8]). Zudem verzeichnen Burgio und Jennessen einen Rückgang der Häufigkeit von onkologischen Diagnosen, während die Daten des Deutschen Kinderkrebsregisters seit 40 Jahren eine zunehmende Inzidenz von Krebs bei Kindern (1980: 95/100.000; 2017: 170/100.000) aufzeigen [7]. Die Heilungsraten sind in diesen 40 Jahren deutlich gestiegen. So zeigte sich für die häufigste kindliche Krebserkrankung, die akute lymphatische Leukämie, eine Gesamtüberlebensrate nach 5 Jahren von 94,7 ± 1,1 % (in der Standardrisiko-Gruppe der ALL-BFM-2009-Studie [9]). Die höheren Überlebensraten wirken sich zusätzlich auf eine Zunahme der Prävalenz aus, die sich in den Daten von Burgio und Jennessen jedoch nicht widerspiegelt.

Laut Burgio und Jennessen (Studienbericht, [8]) ist die Prävalenz bei onkologischen Erkrankungen in der Altersgruppe 19 bis < 20 Jahre am höchsten (201/100.000); die Altersgruppe 15–19 Jahre weist die zweithöchste Prävalenz auf (178/100.000). Dies entspricht nicht den Daten des Deutschen Kinderkrebsregisters zur Altersverteilung von Kindern mit Krebs in Deutschland. Demnach bilden Kleinkinder (und nicht Jugendliche oder junge Erwachsene) die größte Altersgruppe der Neuerkrankungen. Da die Prognose einer kindlichen Krebserkrankung in der Regel in den ersten Jahren nach Diagnosestellung entschieden wird, ist bei Burgio und Jennessen nicht nachvollziehbar, dass die beiden größten Altersgruppen mit onkologischen, lebensbedrohlichen oder lebensverkürzenden Diagnosen junge Erwachsene und Jugendliche bilden sollen. Burgio und Jennessen weisen in der Diskussion zwar auf die Unterschiede zu den Daten des Kinderkrebsregisters hin und erwähnen überlappende Konfidenzintervalle, die damit die Signifikanz und klinische Bedeutung der aufgeführten Unterschiede zum Kinderkrebsregister relativieren würden. Bedauerlicherweise werden die erwähnten Konfidenzintervalle aber weder in der Publikation noch im Studienbericht [8] aufgeführt und verglichen.

Ferner könnte mit der Nennung von jährlichen Neuerkrankungen (letzter Abschnitt der Diskussion vor Limitationen der Studie) bei Burgio und Jennessen der Eindruck entstehen, dass die onkologischen Fälle nur einen geringen Teil in der Analysegruppe darstellen (ca. 2.000 Neuerkrankungen/Jahr im Vergleich zu einer Gesamtprävalenz von > ca. 300.000–400.000) aller PraeKids-Fälle. In Anhang V des Studienberichts [8] findet sich die genaue Zahl von 24.797 onkologischen Diagnosefällen (GKV 2019), die damit 12,9 % aller Fälle der TfSL-Gruppe 1 (Together for Short Lives, Diagnosegruppierung) ausmachen. Weder der Publikation noch dem Studienbericht lassen sich die Aufschlüsselung der einzelnen onkologischen Diagnosen entnehmen (z. B. C91.00 = akute lymphatische Leukämie ALL als die häufigste Krebserkrankung im Kindesalter). Bei einer Offenlegung der onkologischen Einzeldiagnosen wäre überprüfbar gewesen, ob die Verteilung der Diagnosen innerhalb des Studienkollektivs von Burgio und Jennessen mit der Verteilung kindlicher Krebsdiagnosen in Deutschland übereinstimmen würde.

Die in Anlage V des Studienberichts [8] genannte Zahl aller Krebsdiagnosen (nach ICD-10 mit dem Buchstaben „C“; = 24.797) hat bei einer jährlichen mittleren Neuerkrankungszahl von ca. 2.000 Kindern zur Folge, dass mindestens 12 Jahre lang kein Kind an einer Krebserkrankung hätte sterben dürfen, um die Zahl von 24.797 Fällen zu erreichen. Bezieht man die Gesamtprognose/Überlebensrate 5 Jahre nach Erstdiagnose von Krebs bei Kindern in Höhe von etwa 80 % mit ein, bedeutet dies, dass in den Rohdaten der PraeKids-Studie Kinder und Jugendliche enthalten sind, die 15,5 Jahre oder länger nach Diagnose einer Krebserkrankung noch als lebensbedrohlich/lebenslimitierend erkrankter „Burgio-Jennessen-Fall“ dokumentiert werden, obwohl sie seit vielen Jahren als geheilt gelten. Folglich ist anzunehmen, dass bei Burgio und Jennessen eine Vielzahl von Fällen mitgezählt wurde, die nicht (mehr) lebensbedrohlich oder lebenslimitierend erkrankt sind. Die hohe und nicht plausible Zahl Jugendlicher bzw. junger Erwachsener mit einer angeblich lebensbedrohlichen Krebserkrankung ist ein Argument gegen die Verwendung ambulanter Abrechnungsdaten, wie sie von den Autor:innen der PraeKids-Studie gewählt wurde. Im ambulanten Abrechnungswesen setzen z. B. „Chronikerpauschalen“ (EBM-Abrechnungsziffern: 03220 bis 03222; [10]) Anreize, die ICD-10-Diagnosen aus der Vorgeschichte weiter zu übernehmen.

## Filterung der Daten mit verschiedenen Listen von ICD-10-Codes

Die Selektion von Krankheitsfällen mittels einer Liste von ICD-Codes beruht auf dem von Fraser mehrfach verwendeten Ansatz [1–3]. Bei Burgio und Jennessen wird ein Mixed-Methods-Ansatz erwähnt, der über verschiedene Rückkoppelungsschleifen zu einer adaptierten Fraser-Liste und letztlich einer Burgio-Jennessen-Liste geführt habe.

Bei der adaptierten Fraser-Liste wurden insgesamt 12 der 4-stelligen ICD-10-Codes ausgeschlossen (vgl. Burgio und Jennessen, Tab. 1). Bei Burgio und Jennessen wurden weder die der Burgio-Jennessen-Liste zugefügten Codes noch die gesamte Liste (z. B. als Anlage) publiziert. Sie verweisen stattdessen auf den ausführlichen Studienbericht [8], dem diese Informationen entnommen werden könnten. Aus dem Studienbericht geht hervor, dass 67 3-stellige bzw. 165 4‑stellige ICD-10-Codes der Fraser-Liste zugefügt wurden, sodass daraus die Burgio-Jennessen-Liste entstand. Dabei ist nicht zu entnehmen, welche Argumente zur Erweiterung der Fraser-Liste geführt haben. Dies wäre von besonderem Interesse gewesen – insbesondere da Burgio und Jennessen von unterschiedlichen Einschätzungen der befragten 7 nicht namentlich genannten Palliativmediziner:innen berichteten.

Es liegt in der Natur der Sache, dass ein Zuwachs an ICD-Codes zu gestiegenen Fallzahlen und einer höheren Prävalenz führt. Wir hätten uns gewünscht, dass gerade die einzelnen Häufigkeiten der zusätzlichen ICD-10-Codes von Burgio und Jennessen genannt und kritisch diskutiert worden wären. Dies wäre für die Nachvollziehbarkeit der Ergebnisse der PraeKids-Studie hilfreich gewesen.

Der ICD-Code E74 („Sonstige Störungen des Kohlenhydratstoffwechsels“) wurde in die Burgio-Jennessen-Liste übernommen (GKV 2019; Fallzahl: 50.492, d. h. doppelt so viele Fälle wie alle onkologischen Diagnosen zusammen) und in die TfSL-Gruppe 2 (= nicht heilbar) klassifiziert. Dieser Code entspricht bei einer Gesamtgröße der TfSL-Gruppe 2 von 127.243 (GKV 2019) einem Anteil von 39,7 %. Die Autor:innen diskutieren zwar, dass innerhalb des Codes E74 verschiedene Erkrankungen subsumiert werden. Die potenziell bedrohlichen Störungen des Galaktosestoffwechsels (Galactokinasemangel, sog. klassische Galaktosämie; Inzidenz: 1:40.000–1.100.000) sind aber deutlich seltener als die nicht lebensbedrohliche oder lebenslimitierende intestinale Fruktoseintoleranz (Prävalenz ca. 10–25 % in der westlichen Gesamtbevölkerung), die unter dem gleichen Code verschlüsselt werden kann. Hier wäre es hilfreich gewesen, die einzelnen 4‑stelligen Codierungen zu verwenden, um die verzerrenden Effekte besser einschätzen und ggf. ausschließen zu können. Die Angabe von Konfidenzintervallen (wie in den Arbeiten von Fraser et al.) wäre ebenso hilfreich gewesen. Bezüglich des Codes „Sonstige Störungen des Kohlenhydratstoffwechsels“ kann die Hypothese formuliert werden, dass eine ambulante E74 sich erheblich von einer stationären E74 unterscheiden dürfte.

Der 3‑stellige ICD-Code D48 (= „Neubildung unsicheren Verhaltens“) wurde laut Anlage V im Jahr 2019 (7) 38.403-mal detektiert und umfasst damit 13.000 Fälle mehr als die Summe aller onkologischen Diagnosen. Das würde bedeuten, dass Neubildungen unsicheren Verhaltens gänzlich lebenslimitierend und/oder lebensbedrohlich wären und ca. 1,5-mal häufiger wären als alle Krebserkrankungen bei Kindern in Deutschland.

Ferner wurden die ICD-Codes B34.2/​B97.2 (= „Corona-Infektion“) neu in die Burgio-Jennessen-Liste aufgenommen. Diese Erkrankung verläuft jedoch bei Kindern und Jugendlichen in den allermeisten Fällen weder lebensbedrohlich noch lebenslimitierend. Todesfälle durch Corona-Infektionen sind bei Kindern sehr selten. Am 17.02.2022 publizierte die Deutsche Gesellschaft für Pädiatrische Infektiologie (DGPI) eine Studie zu Sterbefällen bei hospitalisierten Kindern und Jugendlichen, die mit einer Corona-Infektion assoziiert waren. Die Zahl der Kinder, die an einer Corona-Infektion verstarben, betrug demnach 22 [11]. Bis zum 21.03.2023 wurden bei Kindern bis 14 Jahren offiziell 5,43 Mio. Corona-Infektionen registriert, sodass die glücklicherweise geringe Mortalität einer Corona-Infektion in den Berechnungen von landesweiten Prävalenzen von lebenslimitierenden oder lebensbedrohlichen Erkrankungen zu vernachlässigen ist [12]. Wären die Jahre 2020 bis 2022 in die PraeKids-Studie einbezogen worden, wären nach der Burgio-Jennessen-Liste Prävalenzen von ca. 10.000–20.000/100.000 nur für die Corona-positiven Kinder festzustellen gewesen.

Schließlich greifen Burgio und Jennessen selbst die Frage nach der Mortalität der gefilterten Erkrankungsfälle auf. In der Diskussion wird von den Autor:innen geschrieben: „Des Weiteren wurden in der Prävalenzberechnung aus Deutschland auch lebensbedrohliche Erkrankungen berücksichtigt, die nicht zwingend zu einem frühen Tod und einer palliativen Versorgung führen müssen (6).“ Die Zahl „6“ verweist auf eine Fußnote, unter der zu lesen ist „schraffiert dargestellt in Abb. 4“. In der Abb. 4 findet man für die TfSL-Gruppe 1 einen großen (ca. 80 % der Fläche?) und in der TfSL-Gruppe 3 einen sehr kleinen (ca. 10 % der Fläche?) schraffierten Bereich. Es finden sich weder in der Publikation noch im Studienbericht Quellen oder Daten, die die schraffiert dargestellten Überlebens- oder Heilungsraten der besagten TfSL-Gruppen zitieren oder belegen würden.

Wir stimmen der Einschätzung der Verantwortlichen der Studie zu, dass aus den Ergebnissen der PraeKids-Studie keine Schlussfolgerungen hinsichtlich der Bedarfe an Kinderhospiz- oder Palliativversorgung abgeleitet werden können. Hierzu sind Studien unter Einbeziehung anerkannter Expert:innen aus der Kinderhospiz- und Palliativversorgung notwendig.
